# Supplementary material for: Negative Regulation of ULK1 by microRNA-106a in Autophagy Induced by a Triple Drug Combination in Colorectal Cancer Cells In Vitro
Source: Genes (Basel). 2021 Feb 9;12(2):245. doi: 10.3390/genes12020245 (PMC7915601; doi:10.3390/genes12020245)
Supplement: Supplementary file 1 [file genes-12-00245-s001.zip › Supplementary Figure 4.pdf]

Supplementary Materials:

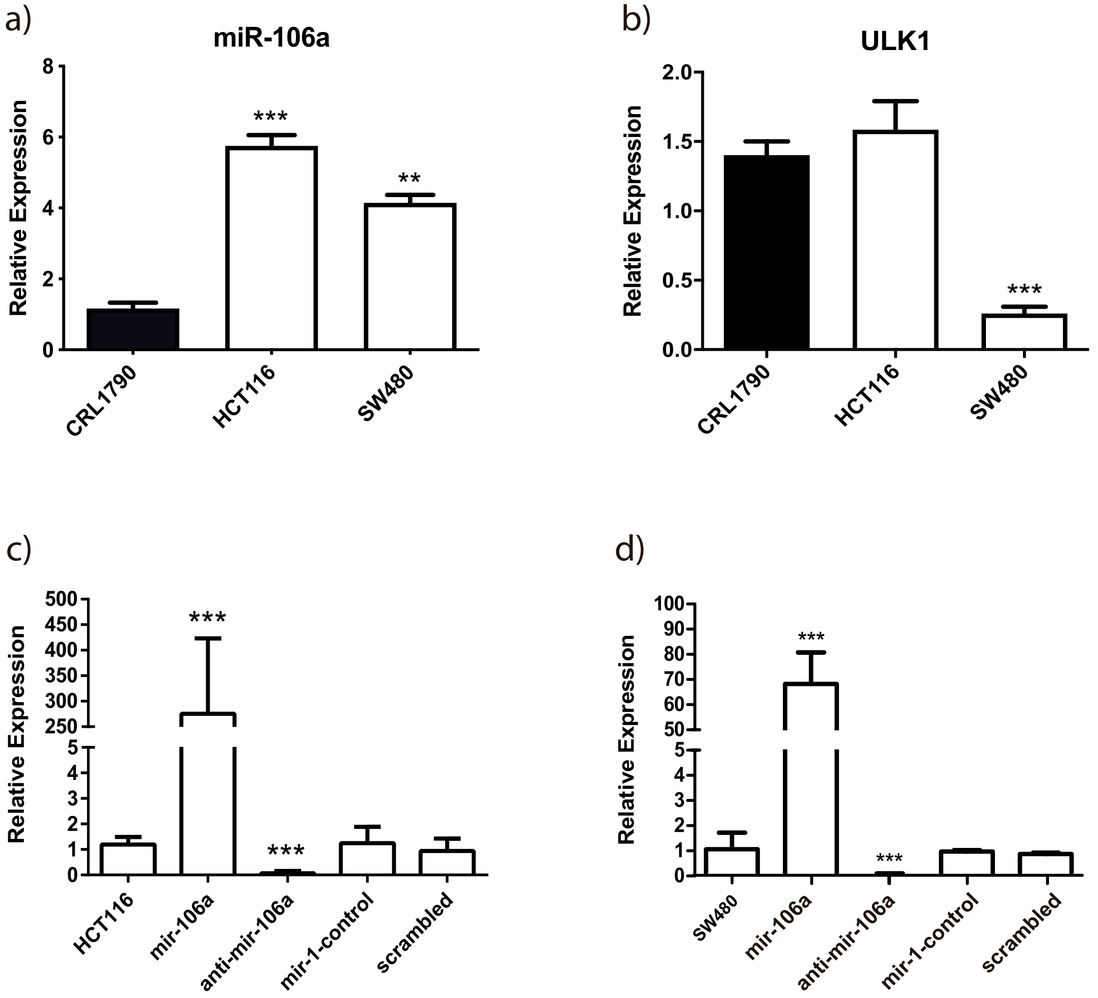

Figure S4. a) and b) Expression of mir-106a and ULK1 in HCT116 and SW480 (CRC cell lines) compared with CRL1790 (normal cells), normalized with RNU6 or GAPDH, respectively. ***p<0.001, **p<0.01. c) and d) Successful transfection of mir-106a mimic, inhibitor and controls was detected by means of qPCR in HCT116 and SW480 cells. ***(p<0.001).
