# Supplementary material for: Negative Regulation of ULK1 by microRNA-106a in Autophagy Induced by a Triple Drug Combination in Colorectal Cancer Cells In Vitro
Source: Genes (Basel). 2021 Feb 9;12(2):245. doi: 10.3390/genes12020245 (PMC7915601; doi:10.3390/genes12020245)
Supplement: Supplementary file 1 [file genes-12-00245-s001.zip › Supplementary Figure 2.pdf]

Supplementary Materials

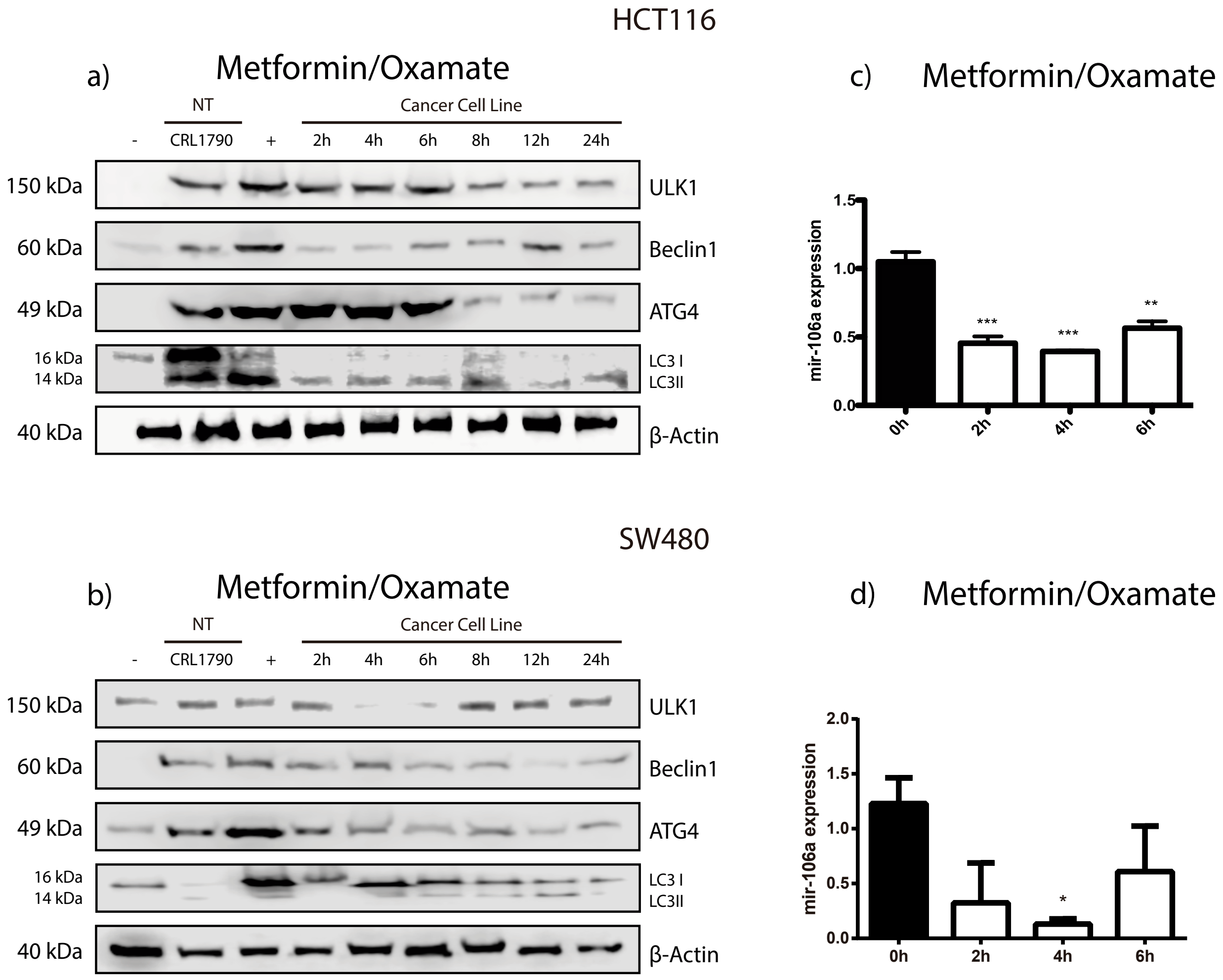

Figure S2. Detection of the proteins involved in the autophagy process in CRL1790 (non-tumor cell line) and HCT116 and SW480 cell lines (negative control=basal cells); positive control rapamycin was used (10μM). Western blot analysis was performed to measure autophagic flux at 2, 4, 6, 8, 12 and 24 hours of exposure a) and b) Metformin/Oxamate. β-actin was used as loading control. Correlation of miR-106a expression with the protein level expression of ULK1 in the HCT116 and SW480 cell lines. Quantitative real- time PCR shows a slight decrease in miR-106a in exposure to c) and d) Metformin/Oxamate. MiR-106a levels were calculated through the ΔΔCt method and normalized relative to U6 snRNA. Data are presented as means ± SD. *$p < 0.05$, **$p < 0.01$, ***$p < 0.001$, respectively.
