# Supplementary material for: Negative Regulation of ULK1 by microRNA-106a in Autophagy Induced by a Triple Drug Combination in Colorectal Cancer Cells In Vitro
Source: Genes (Basel). 2021 Feb 9;12(2):245. doi: 10.3390/genes12020245 (PMC7915601; doi:10.3390/genes12020245)
Supplement: Supplementary file 1 [file genes-12-00245-s001.zip › Supplementary Figure 3.pdf]

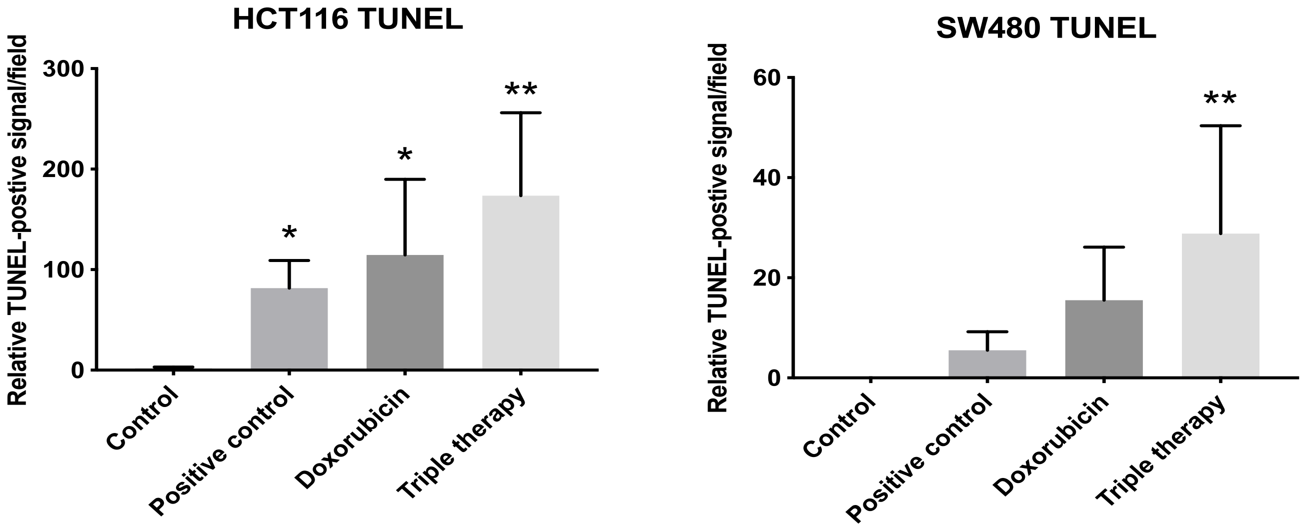

Figure S3. Statistical results of TUNEL. TUNEL-positive cells in HCT116 and SW480 were evaluated by using ImageJ software. *$p < 0.05$, **$p < 0.01$.
