# Supplementary material for: Negative Regulation of ULK1 by microRNA-106a in Autophagy Induced by a Triple Drug Combination in Colorectal Cancer Cells In Vitro
Source: Genes (Basel). 2021 Feb 9;12(2):245. doi: 10.3390/genes12020245 (PMC7915601; doi:10.3390/genes12020245)
Supplement: Supplementary file 1 [file genes-12-00245-s001.zip › Supplementary figure 1.pdf]

Supplementary Materials:

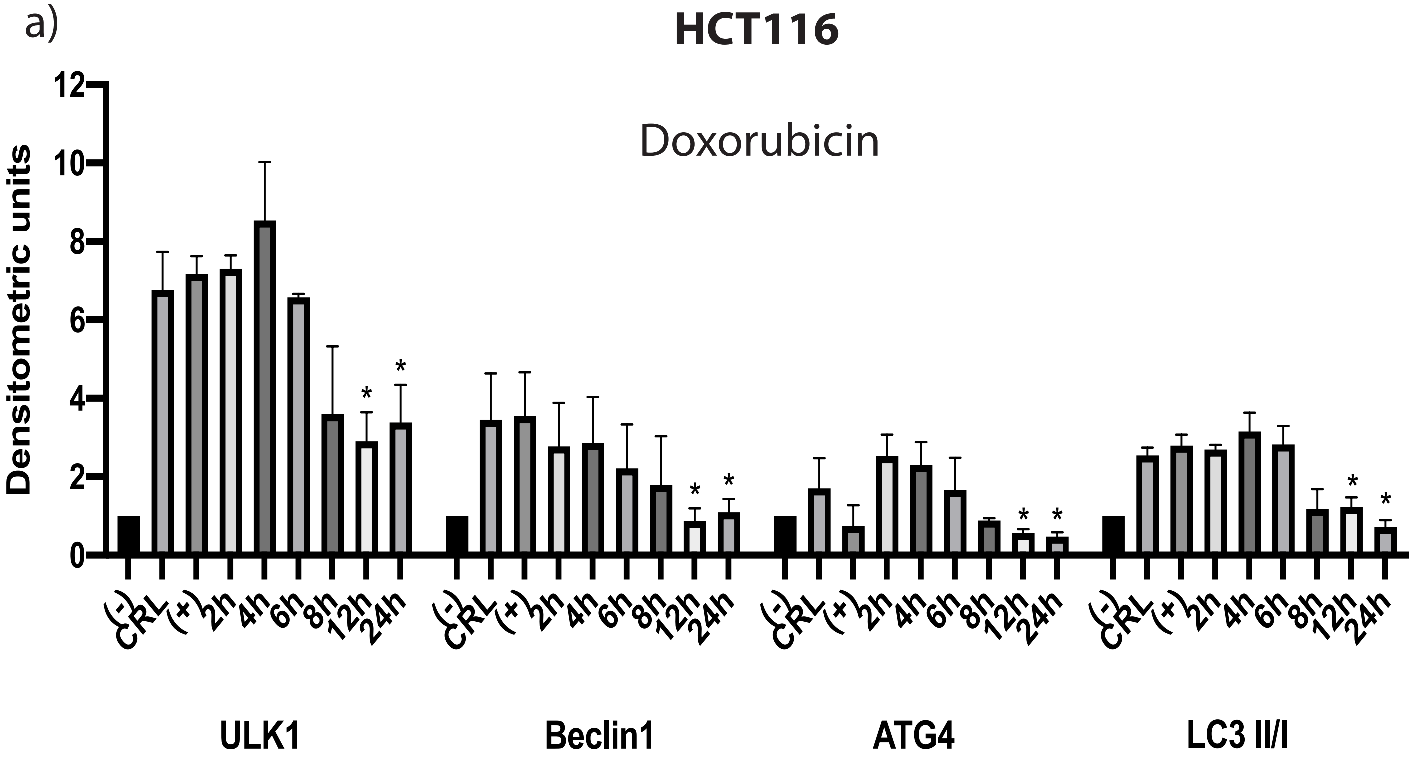

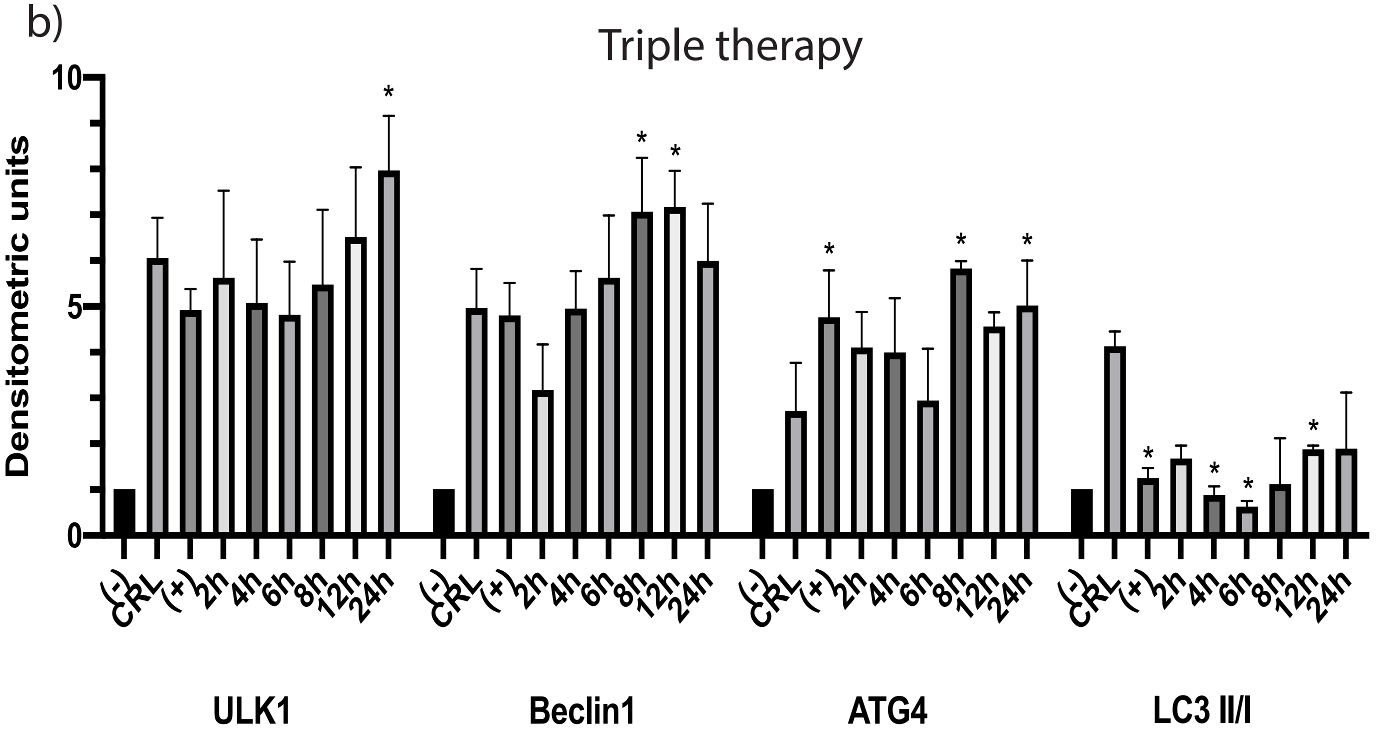

c)

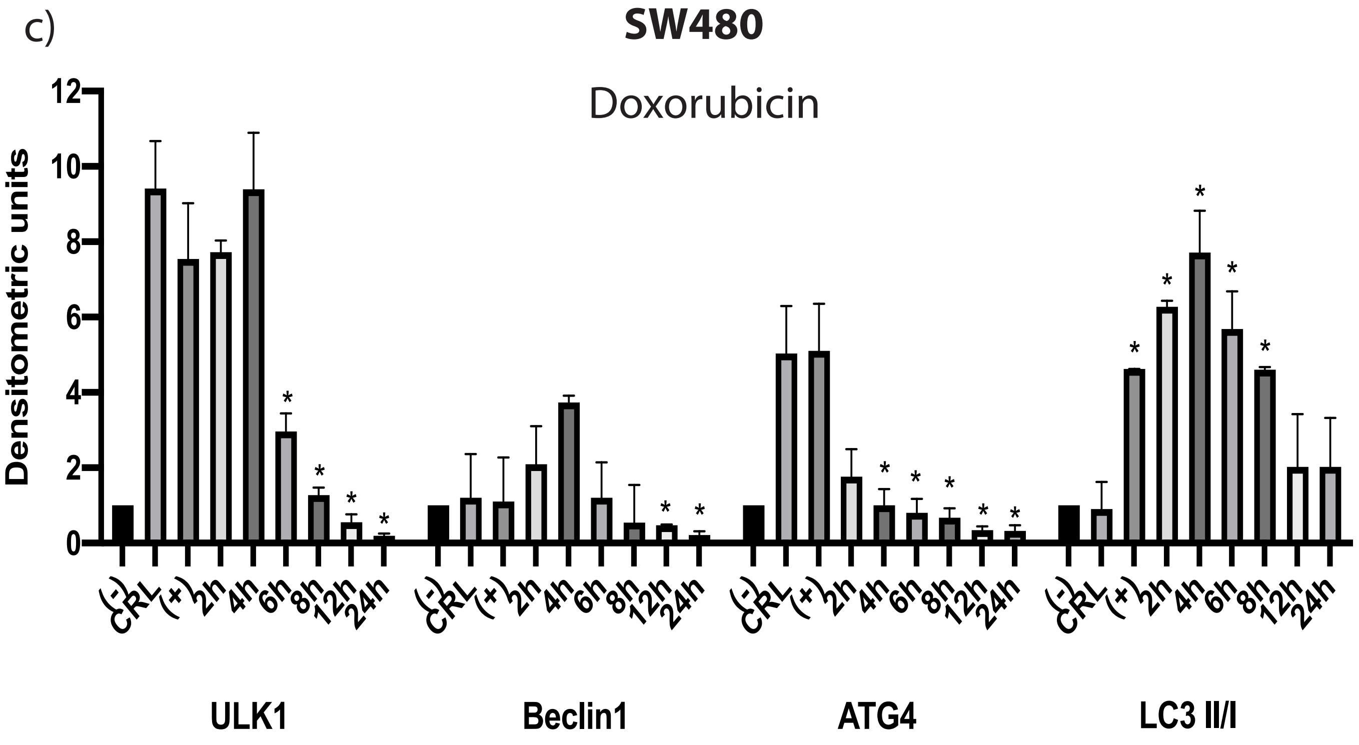
SW480
Doxorubicin
Densitometric units
0
2
4
6
8
10
12
(-)
CRL
(+)
2h
4h
6h
8h
12h
24h
ULK1
Beclin1
ATG4
LC3 II/I

d)

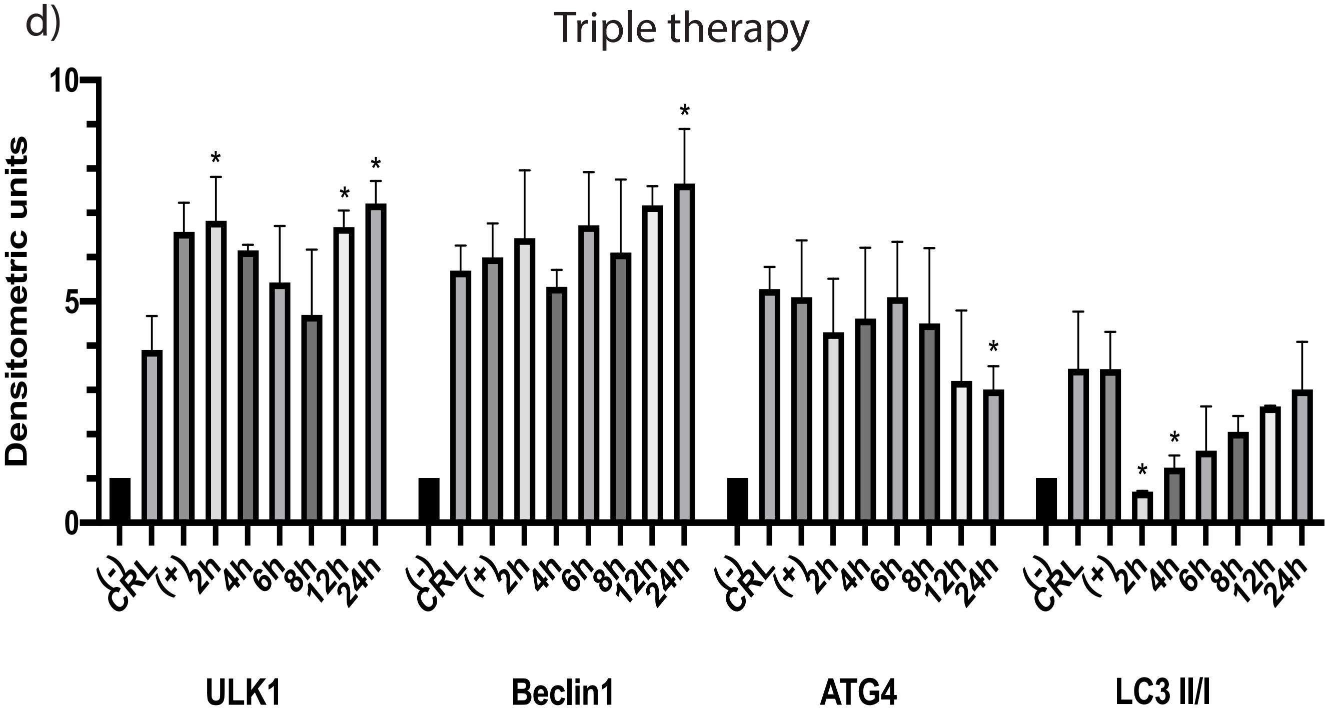
Triple therapy
Densitometric units
0
5
10
(-)
CRL
(+)
2h
4h
6h
8h
12h
24h
ULK1
Beclin1
ATG4
LC3 II/I

Figure S1. Densitometry graphs of the proteins involved in autophagy in HCT116 and SW480 CRC cell lines. a) and c) Doxorubicin: b) and d) triple therapy. Data are presented as means ± SD. *$p<0.05$. Cells treated with doxorubicin decreases overtime, while the detection of the same proteins increases after treated with triple therapy.
